# Engineered hydrogel reveals contribution of matrix mechanics to esophageal adenocarcinoma and identifies matrix-activated therapeutic targets

**DOI:** 10.1172/JCI168146

**Published:** 2023-12-01

**Authors:** Ricardo Cruz-Acuña, Secunda W. Kariuki, Kensuke Sugiura, Spyros Karaiskos, Eleanor M. Plaster, Claudia Loebel, Gizem Efe, Tatiana Karakasheva, Joel T. Gabre, Jianhua Hu, Jason A. Burdick, Anil K. Rustgi

**Affiliations:** 1 Herbert Irving Comprehensive Cancer Center, Division of Digestive and Liver Diseases, Department of Medicine, Vagelos College of Physicians and Surgeons, Columbia University Irving Medical Center, New York, New York, USA.; 2 Department of Biomedical Engineering and; 3 Department of Materials Science and Engineering, University of Michigan, Ann Arbor, Michigan, USA.; 4 Division of Gastroenterology, Hepatology, and Nutrition, Children’s Hospital of Philadelphia, Philadelphia, Pennsylvania, USA.; 5 BioFrontiers Institute and Department of Chemical and Biological Engineering, University of Colorado, Boulder, Colorado, USA.

**Keywords:** Cell Biology, Oncology, Cancer, Drug therapy, Extracellular matrix

## Abstract

Increased extracellular matrix (ECM) stiffness has been implicated in esophageal adenocarcinoma (EAC) progression, metastasis, and resistance to therapy. However, the underlying protumorigenic pathways are yet to be defined. Additional work is needed to develop physiologically relevant in vitro 3D culture models that better recapitulate the human tumor microenvironment and can be used to dissect the contributions of matrix stiffness to EAC pathogenesis. Here, we describe a modular, tumor ECM–mimetic hydrogel platform with tunable mechanical properties, defined presentation of cell-adhesive ligands, and protease-dependent degradation that supports robust in vitro growth and expansion of patient-derived EAC 3D organoids (EAC PDOs). Hydrogel mechanical properties control EAC PDO formation, growth, proliferation, and activation of tumor-associated pathways that elicit stem-like properties in the cancer cells, as highlighted through in vitro and in vivo environments. We also demonstrate that the engineered hydrogel serves as a platform for identifying potential therapeutic targets to disrupt the contribution of protumorigenic matrix mechanics in EAC. Together, these studies show that an engineered PDO culture platform can be used to elucidate underlying matrix-mediated mechanisms of EAC and inform the development of therapeutics that target ECM stiffness in EAC.

## Graphical abstract

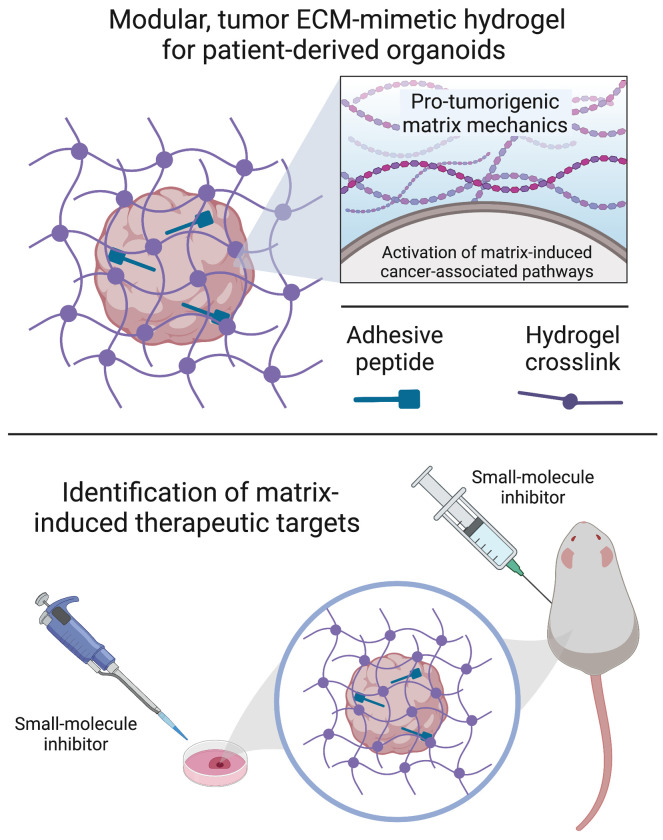

## Introduction

Over the past 30 years, the incidence of esophageal adenocarcinoma (EAC) has risen dramatically, by 300% to 600% in the US (1). Previous studies have demonstrated that changes in the tumor microenvironment involving a stiffened extracellular matrix (ECM) are associated with EAC progression (2–6). Although clinical observations suggest that increased ECM stiffness drives cell transformation, cancer progression, and metastasis, the underlying pathways of mechanotransduction that lead cancer cells to translate mechanical signals into intracellular protumorigenic pathways are yet to be defined (5). Therefore, additional work is needed to develop physiologically relevant 3D culture models that better recapitulate the human tumor microenvironment and can dissect the contributions of matrix properties to elucidate underlying molecular mechanisms of the disease (7).

Patient-derived tumor organoids have become attractive preclinical models for studying cancer biology, as they retain the biological characteristics of the primary tumor (7–9). Indeed, our lab has shown that patient-derived EAC 3D organoids (EAC PDOs) can serve as avatars for studying cellular responses to anticancer drugs, as they recapitulate patients’ drug responses in the clinic (10, 11). Patient-derived organoids (PDOs) are traditionally grown in Matrigel, a heterogeneous, complex mixture of ECM proteins, proteoglycans, and growth factors secreted by Engelbreth-Holm-Swarm mouse sarcoma cells (12). However, Matrigel suffers from lot-to-lot compositional and structural variability and cannot recapitulate the independent role of matrix properties in disease progression due to the inability to uncouple its physicochemical properties (12–14). For instance, making changes to the bulk concentration (e.g., decrease in matrix density) of Matrigel is a common approach for varying its mechanical properties; however, these changes unavoidably alter other matrix properties, such as adhesive ligand density and fiber density/structure (14) (Supplemental Figure 1A; supplemental material available online with this article; https://doi.org/10.1172/JCI168146DS1). Therefore, although modulation of the bulk concentration of Matrigel results in changes in EAC PDO formation (density), growth (area), and transcriptional expression of EAC-associated genes (Supplemental Figure 1, B–D), it is unclear whether this effect is mediated by differences in mechanical or biochemical matrix properties. To address this important gap, well-defined engineered hydrogels are an evolving and important component of tumor PDO culture systems as alternatives to Matrigel, particularly for introducing user-defined microenvironment signals for studying human epithelial tumors (15–19).

Here, we describe a modular, tumor ECM–mimetic hydrogel platform with defined physicochemical properties that support EAC PDO culture and growth. Hydrogel mechanical properties, adhesive ligand presentation, and protease-dependent degradation were key parameters in engineering a hydrogel that supported EAC PDO viability and growth. Particularly, hydrogel mechanical properties controlled EAC PDO formation and growth and activation of tumor-associated pathways. For instance, we showed that increased matrix mechanics enable upregulation of the Yes-associated protein 1 (Yap)/SRY-box transcription factor 9 (Sox9) axis, eliciting stem-like properties in the EAC PDOs and further elucidating an underlying molecular mechanism of the disease. Additionally, the engineered hydrogel served as a platform for identifying potential therapeutic targets for disrupting the contribution of protumorigenic increased matrix mechanics in EAC. Whereas previous work has established engineered hydrogels as tumor ECMs for investigating multicellular assembly and tumor invasion using cancer cell lines (20–22) or studying tumor PDO resistance to therapy (19, 23), we are the first, to our knowledge, to analyze the contributions of ECM mechanical properties to EAC PDO growth, proliferation, and identification of matrix mechanics–mediated drivers of stem-like properties as therapeutic targets through in vitro and in vivo models. Finally, the modular nature of the engineered hydrogel platform allows for potential adaptation to the culture of 3D organoid models of other human cancers. Thus, we provide mechanistic and translational insights with broad applicability.

## Results

### Engineered hydrogel supports EAC PDO development.

**Figure 1:**
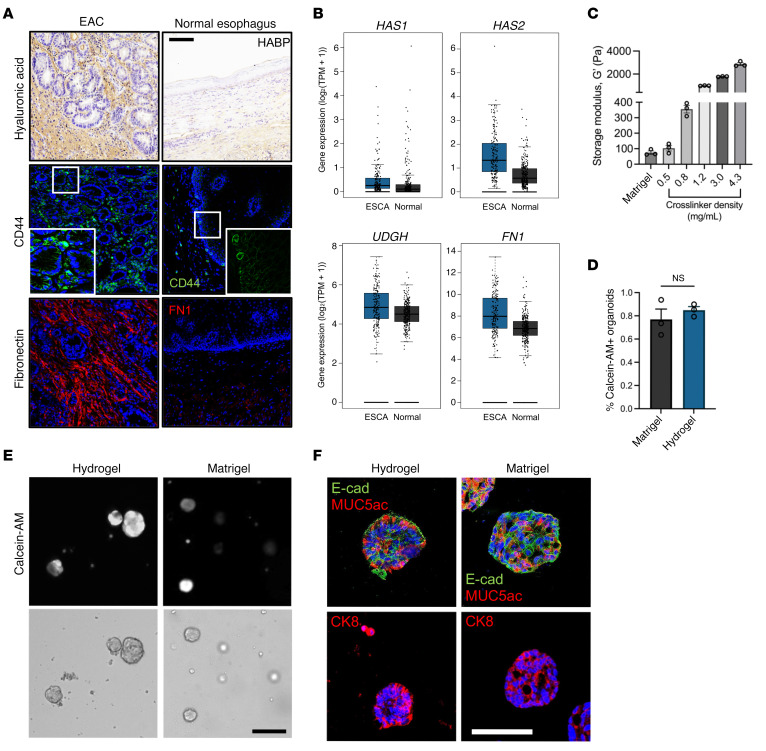
Engineered hydrogel supports EAC PDO development. (**A**) Images of patient tissue sections from normal and EAC biopsies stained for HA (HABP), CD44, or fibronectin (FN1). Scale bar: 100 μm. Original magnification, ×5 (insets). (**B**) Bulk RNA-Seq analysis of ESCA and normal pancreatic tissue samples for fibronectin and hyaluronan-associated genes (HAS1, HAS2, UGDH). *n* = 286 for ESCA; *n* = 283 for normal. (**C**) Relationship between crosslinker density (mg/mL) and storage modulus, G′ (mean ± SEM; *n* = 3 independently prepared hydrogels per condition). (**D**) Quantification of PDO viability as assessed by calcein-AM labeling at 7 days after encapsulation. Viability is quantified as the percentage of PDOs that stained positive for calcein-AM (mean ± SEM; *n* = average number of calcein-AM^+^ organoids per hydrogel; at least 20 organoids per hydrogel were analyzed). Welch’s *t* test with 2-tailed comparison showed no significant differences between groups. NS = *P* > 0.05. (**E**) Representative transmitted light and fluorescence microscopy images of EAC PDOs cultured in NorHA hydrogels or Matrigel. Scale bar: 200 μm. (**F**) Representative fluorescence microcopy images of EAC PDOs within NorHA hydrogels stained for MUC5ac, E-cad, and CK8 at 14 days after encapsulation. Scale bar: 50 μm. Three independent experiments were performed, and data are presented for 1 of the experiments. Every independent experiment was performed with 4 gel samples per experimental group.

We selected a hydrogel platform based on hyaluronic acid (HA), specifically through the crosslinking of norbornene-functionalized HA (NorHA) macromer (Supplemental Figure 2A), which exhibits native biofunctionality and has been extensively developed for in vitro cell activation by stiffening events and several preclinical in vivo applications (24). HA has inherent biological importance due to its binding to cell receptors (e.g., CD44; refs. 25, 26) and is a major component of the tumor niche (Figure 1, A and B), creating a microenvironment that is favorable for tumor angiogenesis, invasion, and metastasis (27, 28). Certainly, EAC patient biopsies showed increased expression of HA in the tumor microenvironment (Figure 1A), whereas RNA-Seq analysis of 286 esophageal carcinoma (ESCA) tissues collected from The Cancer Genome Atlas (TCGA) and The Genome-Tissue Expression Project (GTEx) (29) confirmed increased expression of HA synthesis genes (*HAS1*, *HAS2*, *UDGH*) as compared with 283 normal esophageal tissues (Figure 1B). Moreover, our hydrogel system offers marked advantages due to its well-defined structure, covalent incorporation of peptide sequences for enhanced cell/matrix interactions, and user-defined hydrogel stiffness, which is controlled by varying the crosslinking peptide concentration, which mediates crosslinking via a thiol-norbornene reaction to form a NorHA hydrogel (Figure 1C and Supplemental Figure 2A) (30). Indeed, HA-based hydrogels have been used by other groups to study tumor progression and resistance to therapy of other cancer types (e.g., colorectal and pancreatic adenocarcinomas) (31, 32). We added to this model system tunable matrix properties that enable an advancement in studying ECM-mediated tumor progression using PDOs.

In our study, we explored a NorHA hydrogel formulation that supports the viability of EAC PDOs generated in Matrigel. After EAC PDOs were grown in Matrigel, they were retrieved, dissociated into single cells, and encapsulated in NorHA hydrogels with mechanical properties similar to those of Matrigel (G′ = 100 Pa; Figure 1C and Supplemental Figure 2B). NorHA hydrogels were engineered to present a constant 2.0 mM RGD adhesive peptide (GCGYGRGDSPG) density and crosslinked with the protease-degradable peptide VPM (0.5 mg/mL; GCNSVPMSMRGGSNCG). Incorporation of 2.0 mM RGD adhesive peptide and a protease-degradable crosslinker to engineered hydrogels has previously promoted epithelial organoid development (13, 33, 34). Moreover, the RGD adhesive ligand (α_5_β_1_ and α_ν_β_3_ integrin–binding peptide) is found in many adhesive proteins, including fibronectin, a major ECM protein component in EAC (2, 35). Indeed, EAC patient biopsies showed increased expression of fibronectin as compared with normal tissue (Figure 1A), and RNA-Seq analysis from TCGA and GTEx (29) confirmed increased expression of fibronectin (*FN1*) in ESCA as compared with normal esophageal tissues (Figure 1B). EAC PDOs grown in NorHA hydrogels functionalized with RGD and crosslinked with VPM demonstrated viability comparable to those grown in Matrigel (Figure 1, D and E). However, when EAC PDOs were grown in NorHA hydrogels presenting an inactive scrambled peptide (RDG) or functionalized with RGD and crosslinked with nondegradable agent 1,4-dithiothreitol (DTT), EAC PDOs showed reduced viability at 7 days after encapsulation, as compared with hydrogels functionalized with RGD and crosslinked with VPM (Supplemental Figure 3, A–C). Moreover, PDOs cultured in the engineered hydrogel formulation showed expression of the epithelial marker E-cadherin (E-cad) and of EAC-specific markers mucin 5AC (MUC5ac) and cytokeratin 8 (CK8) at levels similar to those of PDOs cultured in Matrigel (Figure 1F). Finally, to determine whether NorHA hydrogels are suitable for the culture of other human PDOs, we embedded Barrett’s esophagus (a precursor or premalignant condition that predisposes to EAC) PDOs (BE PDOs) (36) in the engineered NorHA hydrogel (Supplemental Figure 3, D–F). BE PDOs cultured in the engineered matrix maintained high viability and comparable growth and formation (density) compared with BE PDOs cultured in Matrigel (Supplemental Figure 3, D–F). Taken together, these data suggest the requirement of specific matrix properties that are essential for organoid viability and formation, establishing the engineered NorHA hydrogel as a culture system for EAC PDOs that has the potential to be adapted for the generation of different human tumor organoids.

### Matrix mechanics control EAC PDO development.

**Figure 2:**
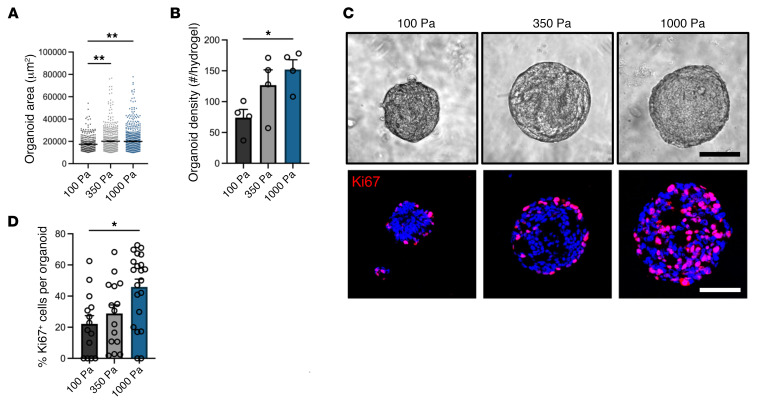
Engineered hydrogel stiffness modulates EAC PDO development. (**A**) Quantification of PDO (**A**) size (area) and (**B**) density as a function of matrix stiffness at 14 days after encapsulation. Data are represented as mean ± SEM. (**A**) *n* = at least 300 organoids analyzed across 4 hydrogels per group; (**B**) *n* = 4 hydrogels per group. (**A** and **B**) Kruskal-Wallis test with Dunn’s multiple-comparisons test showed significant differences between 100 Pa and 350 Pa or 1000 Pa. (**C**) Representative transmitted light and fluorescence microscopy images of EAC PDOs and (**D**) quantification of proliferating cells (%Ki67^+^) in EAC PDOs cultured in NorHA hydrogels of different stiffnesses at 14 days after encapsulation. Data are represented as mean ± SEM. *n* = at least 15 organoids analyzed per group. Kruskal-Wallis test with Dunn’s multiple-comparisons test showed significant differences between 100 Pa and 1000 Pa. Scale bar: 100 μm. (**A**–**D**) Three independent experiments were performed, and data are presented for 1 of the experiments. Every independent experiment was performed with 4 gel samples per experimental group. *P < 0.05; **P < 0.01.

As ECM mechanical properties influence epithelial cell behavior (33, 37), we investigated the influence of crosslinker density, which controls hydrogel mechanical properties (Figure 1C), on EAC PDO size, formation, and proliferation (Figure 2). Hydrogels were engineered to present constant NorHA macromer and adhesive ligand densities, but with varying crosslinker densities. EAC PDOs were embedded in NorHA hydrogels with mechanical properties that ranged from a “soft” hydrogel (0.5 mg/mL VPM; G′ = 100 Pa, similar to Matrigel) to a “stiff” hydrogel (1.2 mg/mL; G′ = 1000 Pa, similar to tumor ECM) and cultured for 14 days. Importantly, the mechanical properties of our stiff hydrogel have been shown to promote protumorigenic behavior in normal cells in previous in vitro studies (38, 39) and compare favorably with measurements of human tumor stiffness (G′ ≥ 1,000 Pa) (38, 40–42). EAC PDOs embedded in stiff (G′: 1,000 Pa) NorHA hydrogels showed significant increases in the size (area) and formation (density) of organoids formed per hydrogel as a function of matrix stiffness (Figure 2, A–C). Similarly, PDOs embedded in the stiff hydrogel condition showed increased cell proliferation, as compared with organoids embedded in the soft hydrogel condition (Figure 2, C and D). Interestingly, when we embedded cells in “stiffer” NorHA hydrogels, namely of G′ = 1,800 Pa and 2,800 Pa, the organoids showed significant reduction in formation, growth, and cell proliferation or no instances of organoid formation, respectively, as compared with the stiff (G′ = 1,000 Pa) NorHA hydrogel (Supplemental Figure 4). Moreover, the engineered hydrogel was able to support culture and expansion of 3 different EAC PDO lines for at least 3 passages (~1.5 months), and after the long-term culture, all EAC PDO lines maintained a significant increase in PDO formation (density), size (area), and cell proliferation as a function of matrix stiffness (Supplemental Figure 5). Together, these data demonstrate that the NorHA hydrogel supports robust long-term in vitro culture and expansion of PDOs and that a restricted range of matrix stiffness (G′ = 1,000 Pa) nurtures EAC PDO growth, formation, and proliferation. These observations establish the engineered hydrogel system as an innovative platform for investigating the independent contributions of matrix mechanics in EAC PDO development.

### Matrix mechanics modulates YAP activation in EAC PDOs.

**Figure 3:**
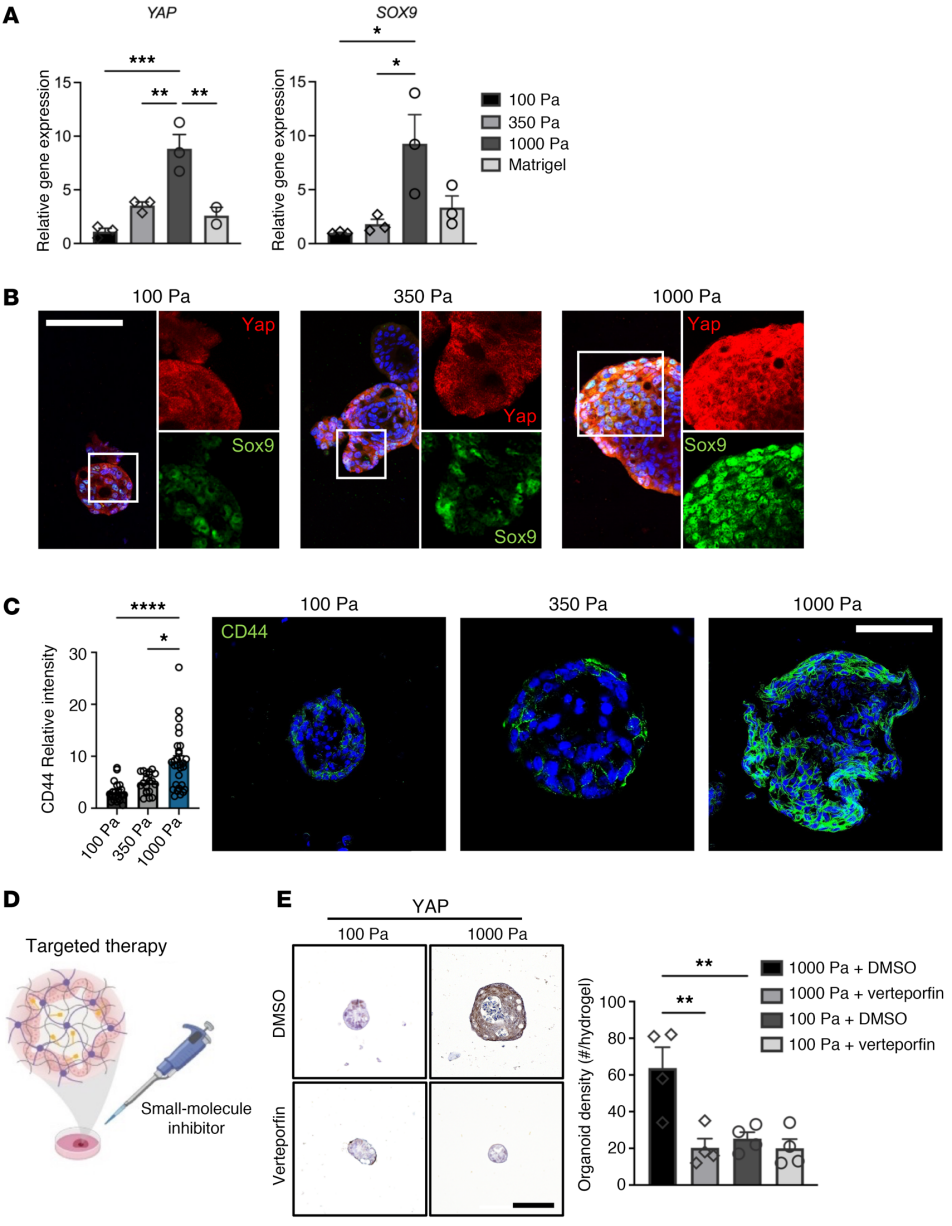
Engineered hydrogel stiffness modulates YAP activation in EAC PDOs. (**A**) Transcriptional expression and (**B**) representative fluorescence images of YAP and SOX9 in organoids within NorHA hydrogels of different stiffness at 14 days after encapsulation. Data are represented as mean ± SEM. *n* = 3 technical replicates, representative of 3 independent experiments. (**A**) One-way ANOVA with Tukey’s multiple comparisons test showed significant differences between 100 Pa and 1000 Pa, 350 Pa and 1000 Pa, and 1000 Pa and Matrigel. *P < 0.05; **P < 0.01; ***P < 0.001. RNA levels normalized to 100 Pa. Scale bars: 100 μm. Original magnification, ×5 (insets, 100 Pa and 350 Pa); ×3 (insets, 1,000 Pa). (**C**) Quantification and representative fluorescence microscopy images of CD44 expression in EAC PDOs cultured in NorHA hydrogels of different stiffness at 14 days after encapsulation. Data are represented as mean ± SEM. *n* = at least 20 organoids analyzed per group. Kruskal-Wallis test with Dunn’s multiple-comparisons test showed significant differences between 100 Pa and 1000 Pa, and 350 Pa and 1000 Pa. *P < 0.05; ****P < 0.0001. Scale bar: 100 μm. (**D**) Schematic of in vitro experiment of EAC PDOs within NorHA hydrogels being treated with YAP inhibitor verteporfin. Created with BioRender.com. (**E**) Representative immunohistochemistry microscopy images and quantification of Yap expression in EAC PDOs cultured in NorHA hydrogels of different stiffnesses at 7 days after encapsulation and treated with 5 nM verteporfin or DMSO. Data are represented as mean ± SEM. *n* = 4 hydrogels per group. One-way ANOVA with Tukey’s multiple-comparisons test showed significant differences between 1000 Pa+DMSO and every other group (**P < 0.01), and no significant differences among other groups (*P* > 0.05). Scale bar: 100 μm. (**A**–**E**) Three independent experiments were performed, and data are presented for 1 of the experiments. Every independent experiment was performed with 4 gel samples per experimental group. *P < 0.05; **P < 0.01; ***P < 0.001; ****P < 0.0001.

Recent work has demonstrated that dysregulated Yap activation is essential for the growth of most solid tumors, acting by inducing cancer stem cell features, proliferation, and metastasis (43–45). Dysregulated Yap activation is a major determinant of stem cell properties by direct upregulation of SOX9 (46, 47) in EAC. However, the pathophysiological event or events that elicit upregulation of the Yap/Sox9 axis in EAC remain elusive. Therefore, as Yap functions as a sensor of the structural and mechanical features of the cellular microenvironment, we investigated whether changes in matrix biomechanics played a role in the expression of Yap and Sox9 in EAC PDOs. EAC PDOs embedded in the stiff hydrogel showed a significant increase in the expression and nuclear localization of Yap as well as SOX9 expression as compared with the 3D organoids within the softer hydrogels or Matrigel (Figure 3, A and B, Supplemental Figure 4B, and Supplemental Figure 6, A and B). This phenotype was also observed in our 3 EAC PDO lines after long-term culture (Supplemental Figure 5, D–F). Moreover, the protein expression of the esophageal cancer putative stem cell marker CD44 (48, 49) was significantly higher in EAC PDOs within the stiff hydrogel as compared with 3D organoids within the softer hydrogels (Figure 3C). These data suggest that matrix mechanics induce aberrant activation of the Yap/Sox9 axis, endowing stem-like properties to the EAC PDOs, as evidenced by increased PDO growth, formation, cell proliferation, and CD44 expression in 3D organoids embedded in the stiff (G′ = 1,000 Pa) NorHA hydrogel.

The expression of other EAC-associated genes, *TP53* and *STAT3*, significantly increased in EAC PDOs within the stiff hydrogel as compared with the 3D organoids within the soft hydrogel (Supplemental Figure 6, C–E). Interestingly, whole-exome sequencing (WES) of the EAC PDOs cultured in NorHA hydrogels of different stiffness (100 or 1000 Pa) or Matrigel revealed the presence of a *TP53* gene Pro72Arg (rs1042522) single-nucleotide polymorphism, which is located in the p53 proline-rich domain and has been associated with increased tumor metastasis (Supplemental Figure 6F) (50). Further analysis revealed that the overall mutational profile of the PDOs, as well as the type and number of *TP53* variations or mutations, did not change as a function of matrix type (Matrigel versus NorHA) or matrix stiffness (100 Pa versus 1000 Pa NorHA) within a 14-day time period (Supplemental Figure 6, F and G). These data demonstrate that the engineered hydrogel can serve as a platform for studying the influence of matrix mechanics in the activation of regulatory mechanisms in EAC PDOs. Surprisingly, bulk RNA-Seq of EAC PDOs did not reveal differentially expressed genes in the 3D organoids within the stiff hydrogel as compared with 3D organoids within the soft hydrogel. However, when comparing EAC PDOs within the stiff hydrogel versus Matrigel, analyses revealed 424 differentially expressed genes (224 upregulated and 200 downregulated genes). While *YAP* was upregulated, this was not statistically significant (Supplemental Figure 7A). Among the top 10 significantly upregulated genes, 5 have been associated with tumor progression, metastasis, or recurrence (*CALB2*, ref. 51, *ECM1*, refs. 52, 53; *TNIK*, ref. 54; *IGFBP4*, ref. 55; and *TCN1*, ref. 56); Supplemental Figure 7B), including *TNIK* (57) and *IGFBP4* (58), which are reported downstream targets of Yap. Finally, gene ontology of up- and downregulated genes showed enrichment of biological processes that have been associated with tumor pathobiology (Supplemental Figure 7C).

Another advantage of HA-based hydrogels is their permissive diffusional properties, which allow diffusion of small molecules, including drugs and inhibitors, to cells (Figure 3D) (24, 59). Therefore, we investigated to determine whether the effect of increased matrix stiffness on EAC PDOs was repressed via inhibition of the nuclear translocation of Yap using verteporfin, a commercially available small molecule inhibitor whose efficacy and potential as therapy has been described previously (60, 61). Addition of verteporfin to the cell culture media of EAC PDOs grown in NorHA hydrogels (Figure 3D) resulted in significant reduction in Yap expression and the formation (density) and size of organoids within the stiff hydrogel, as compared with the vehicle control (DMSO) (Figure 3E and Supplemental Figure 8A). However, EAC PDOs embedded in the soft hydrogel and exposed to verteporfin showed no significant differences in Yap expression, organoid formation, and size as compared with vehicle control (Figure 3E and Supplemental Figure 8A). Similarly, introduction of YAP siRNA via lipofection to the EAC PDOs grown in the stiff NorHA hydrogel resulted in significant reduction in 3D organoid formation (density), size (area), Yap and Sox9 expression, and Yap nuclear localization as compared with the control siRNA (Supplemental Figure 8, B–E). However, EAC PDOs embedded in the soft hydrogel with the addition of Yap siRNA showed no significant differences in 3D organoid formation, size, Yap and Sox9 expression, and Yap nuclear localization as compared with control siRNA (Supplemental Figure 8, B–E). These complementary data further underscore that matrix mechanics modulate the activation of the Yap/Sox9 axis. Together, these data elucidate a mechanism showing how matrix mechanics influence EAC pathogenesis and nominate YAP as a potential therapeutic target in this context.

### Matrix mechanics control EAC PDO development and YAP activation in vivo.

**Figure 4:**
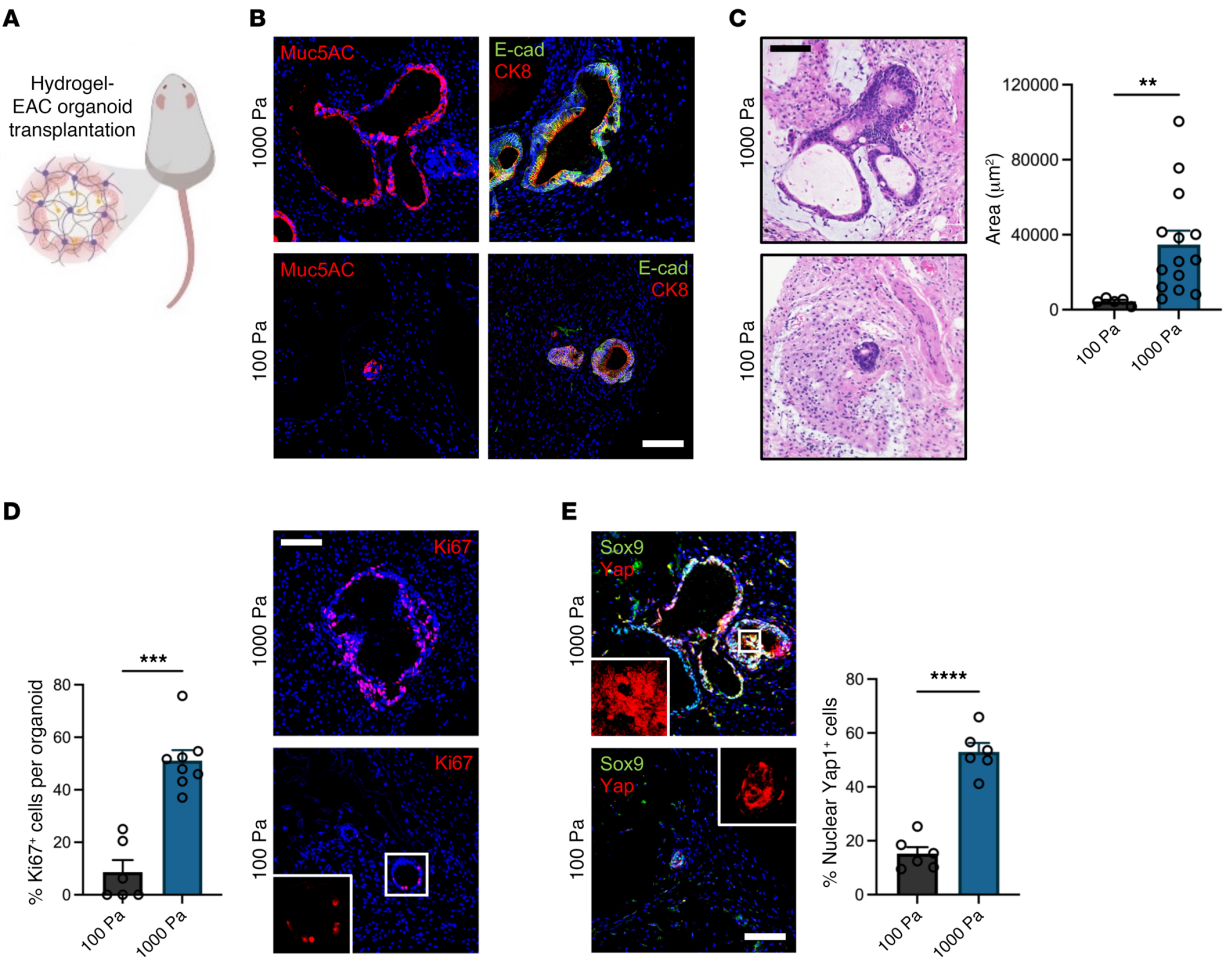
Engineered hydrogel stiffness-dependent growth of EAC PDOs in in vivo xenograft model. (**A**) Schematic of in vivo transplantation experiment of EAC PDOs within NorHA hydrogels into mouse subcutaneous pockets. Created with BioRender.com. (**B**) Representative fluorescence microcopy images of EAC PDOs within NorHA hydrogels stained for MUC5ac, E-cad, and CK8 at 28 days after encapsulation and in vivo transplantation. Scale bar: 100 μm. (**C**) Histological (H&E) microcopy images and quantification of PDO size (area) as a function of matrix stiffness at 28 days after encapsulation and in vivo transplantation. Data are represented as mean ± SEM. *n* = at least 5 organoids analyzed per group. Welch’s *t* test with 2-tailed comparison showed significant differences between 100 Pa and 1000 Pa. **P < 0.01. Scale bar: 100 μm. (**D**) Quantification and representative fluorescence microscopy images of percentages of proliferating cells (%Ki67^+^) per EAC PDOs as a function of matrix stiffness at 28 days after encapsulation and in vivo transplantation. Data are represented as mean ± SEM. *n* = at least 6 organoids analyzed per group. Mann-Whitney *U* test showed significant differences between 100 Pa and 1000 Pa. ***P < 0.001. Scale bar: 100 μm. (**E**) Representative fluorescence microcopy images of EAC PDOs within NorHA hydrogels stained for Sox9 and Yap at 28 days after encapsulation and in vivo transplantation. Quantification of percentage of nuclear Yap^+^ cells (%Yap^+^) per EAC PDO as a function of matrix stiffness. Data are represented as mean ± SEM. *n* = 6 organoids analyzed per group. Welch’s *t* test with 2-tailed comparison showed significant differences between 100 Pa and 1000 Pa. ***P < 0.001. Scale bar: 100 μm. Original magnification, ×5 (insets). (**A**–**E**) Two independent experiments were performed, and data are presented for 1 of the experiments. Every independent experiment was performed with 2 gels per mouse and 5 mice per experimental group.

Engineered hydrogels have been utilized previously as organoid delivery vehicles (24, 34). Therefore, we embedded organoids in our engineered tumor ECM-mimetic hydrogels and transplanted into dorsal subcutaneous spaces of immunocompromised mice to study the effects of matrix mechanics (Figure 4A). After 4 weeks, PDOs showed expression of the epithelial marker E-cad and of EAC-specific markers CK8 and MUC5ac (Figure 4B). However, implanted stiff hydrogels contained significantly larger (area) EAC PDOs as compared with organoids within soft hydrogels (Figure 4C). Additionally, PDOs embedded in the stiff hydrogel showed increased cell proliferation, Sox9 expression, and nuclear localization of YAP, as compared with organoids embedded within the soft hydrogel (Figure 4, D and E). These data suggest that matrix mechanics control PDO growth and proliferation via dysregulated activation of the Yap/Sox9 axis in an in vivo environment. Finally, as studies suggest that increased ECM stiffness stabilizes mutant p53 (62) and we have previously shown that mutant p53-Yap interactions promote esophageal cancer progression (63), we investigated p53 expression in EAC PDOs within NorHA hydrogels. We observed that EAC PDOs within the stiff hydrogel show increased nuclear p53 localization as compared with organoids within the soft hydrogel (Supplemental Figure 6H). Interestingly, we did not observe a significant change in Stat3 expression as a function of matrix stiffness in vivo (Supplemental Figure 6I). Together, these data further underscore that matrix mechanics modulate the dysregulated activation of Yap/Sox9 and potentially the expression of other EAC-associated proteins, elucidating underlying mechanisms of EAC in the context of matrix mechanics using an engineered in vivo model.

### Yap inhibition represses the effect of matrix mechanics in EAC PDOs in vivo.

**Figure 5:**
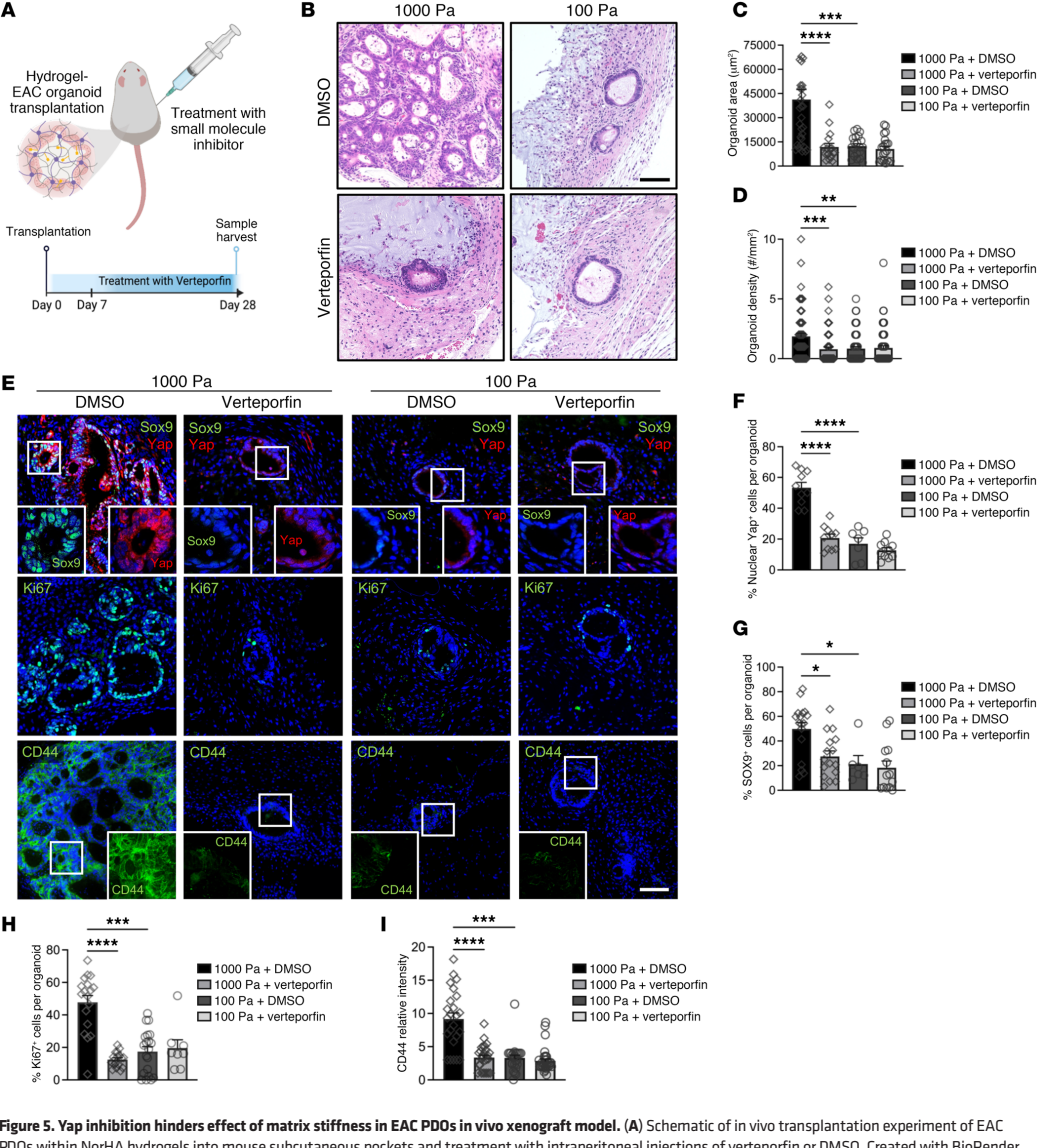
Yap inhibition hinders effect of matrix stiffness in EAC PDOs in vivo xenograft model. (**A**) Schematic of in vivo transplantation experiment of EAC PDOs within NorHA hydrogels into mouse subcutaneous pockets and treatment with intraperitoneal injections of verteporfin or DMSO. Created with BioRender.com. (**B**) Histological (H&E) microcopy images and quantification of PDO (**C**) size (area) and (**D**) density within NorHA hydrogels at 28 days after encapsulation, in vivo transplantation, and treatment with verteporfin or DMSO. Data are represented as mean ± SEM. (**C**) *n* = at least 17 organoids analyzed across 10 samples per group. (**D**) *n* = at least 55 organoids analyzed across 10 samples per group. Kruskal-Wallis test with Dunn’s multiple-comparisons test showed significant differences between 1000 Pa+DMSO and 1000 Pa +verteporfin, 1000 Pa+DMSO and 100 Pa+DMSO and no significant differences between 100 Pa+DMSO and 100 Pa+verteporfin (*P* > 0.05). **P < 0.01; ***P < 0.001; ****P < 0.0001. Scale bar: 100 μm. (**E**) Fluorescence microcopy images of PDOs within NorHA hydrogels at 28 days after encapsulation, in vivo transplantation, and treatment with verteporfin or DMSO stained for Sox9, Yap, Ki67, and CD44. Scale bar: 100 μm. Original magnification, ×5 (insets). (**F**–**I**) Quantification of (**F**) percentage of nuclear Yap^+^ cells (%Yap^+^), (**G**) percentage of Sox9^+^ cells (%Sox9^+^), (**H**) percentage of Ki67^+^ cells (%Ki67^+^), and (**I**) CD44 fluorescence intensity per EAC PDO within NorHA hydrogels at 28 days after encapsulation, in vivo transplantation, and treatment with verteporfin or DMSO. Data are represented as mean ± SEM. (**F**–**I**) *n* = at least 6 organoids analyzed per group. Kruskal-Wallis test with Dunn’s multiple-comparisons test showed significant differences between 1000 Pa+DMSO and 1000 Pa+verteporfin, 1000 Pa+DMSO and 100 Pa+DMSO and no significant differences between 100 Pa+DMSO and 100 Pa+verteporfin (*P* > 0.05). *P < 0.05; ***P < 0.001; ****P < 0.0001. (**A**–**G**) Two independent experiments were performed, and data are presented for 1 of the experiments. Every independent experiment was performed with 2 gels per mouse and 5 mice per experimental group.

Patient-derived xenograft (PDX) models have proven to be highly effective in predicting the efficacy of both conventional and novel anticancer therapeutics (64). Therefore, we exploited the native biocompatibility of the engineered NorHA hydrogel and the susceptibility of EAC PDOs to anticancer drugs (10) and applied an in vivo xenograft model for targeted therapy. EAC PDOs were embedded in soft or stiff NorHA hydrogels and transplanted into the dorsal subcutaneous space of immunocompromised mice. After 1 week, mice were treated with intraperitoneal injections of verteporfin or vehicle control (DMSO) for 3 weeks (Figure 5A). At the end of the treatment, EAC PDOs in stiff hydrogels from mice treated with verteporfin were significantly lower in density and smaller in size as compared with organoids within implanted stiff hydrogels from the control (DMSO) group (Figure5, B–D, and Supplemental Figure 8F). Additionally, PDOs within the stiff hydrogel from mice treated with verteporfin showed a significant decrease in Yap expression and nuclear localization (Figure 5, E and F), Sox9 expression (Figure 5, E and G), cell proliferation (Figure 5, E and H), and CD44 expression (Figure 5, E and I) as compared with organoids within stiff hydrogels from control (DMSO) mice (Figure 5, E–I). Interestingly, EAC PDOs within implanted soft hydrogels from mice treated with verteporfin showed no significant difference in organoid density and size as compared with organoids within implanted soft hydrogels from control (DMSO) mice (Figure 5, B–D, and Supplemental Figure 8F). Concurrently, when comparing PDOs within the soft hydrogel from mice treated with verteporfin to those of control (DMSO) mice, organoids showed no significant difference in Yap expression and nuclear localization (Figure 5, E and F), Sox9 expression (Figure 5, E and G), cell proliferation (Figure 5, E and H), and CD44 expression (Figure 5, E and I). These data suggest that the effect of increased matrix mechanics in EAC PDOs is mediated in part by matrix stiffness–dependent activation of Yap/Sox9 in an in vivo environment, elucidating an underlying mechanism of EAC. These data further suggest that matrix stiffness–mediated activation of Yap/Sox9 in EAC PDOs elicits stem-like properties, resulting in increased organoid formation, growth, proliferation, and CD44 expression in an in vivo environment. Finally, these results suggest that NorHA hydrogels can serve as a platform for the identification of matrix-activated therapeutic targets in patient-derived tumor organoid xenograft studies.

## Discussion

In this study, we have established a tumor ECM–mimetic hydrogel platform with tunable mechanical properties, controlled presentation of cell-adhesive ligands, and protease-dependent degradation that supports the long-term culture and expansion of EAC PDOs. Both mechanical and biochemical properties of the engineered hydrogel were important for enabling organoid formation and viability, and we have identified an optimal formulation that supports EAC PDO survival, growth, and expansion. Presentation of the cell-adhesive ligands and hydrogel susceptibility to protease-dependent degradation was essential for cell viability and organoid formation, consistent with previous work (33). In addition, we demonstrated that this engineered hydrogel formulation supports the development of other PDOs, such as Barrett’s esophagus (BE) organoids. This is important in that BE is a major precursor to EAC, thereby permitting elucidation of how matrix mechanics influence progression from a precancer state to a cancer state. Moreover, hydrogel mechanics had direct control over EAC PDO’s fate, eliciting a stem-like behavior via matrix stiffness–mediated activation of the Yap/Sox9 axis. Specifically, we showed that EAC PDOs cultured in NorHA hydrogels with disease-relevant mechanical properties (G′ = 1000 Pa) had marked increases in organoid formation, growth, proliferation, and activation of tumor-associated pathways, as compared with organoids within softer and stiffer hydrogels. Our observations provide insights into how cancer cells modulate activity of transcription factors that promote cell proliferation and survival in response to a narrow range of ECM stiffness via mechanotransduction pathways. Furthermore, the well-defined engineered hydrogel addresses major limitations of Matrigel associated with lot-to-lot variability and inability to uncouple matrix physicochemical properties. These observations establish the engineered hydrogel as a 3D platform for dissecting the protumorigenic contributions of matrix mechanics to EAC development in in vitro and in vivo settings, thereby addressing a major gap in the field.

Although increased ECM stiffness drives malignant cell transformation, progression, and metastasis, current traditional PDX models do not account for tumor ECM mechanics. Therefore, we also exploited the native biocompatibility of the NorHA hydrogel and the susceptibility of EAC PDOs to anticancer drugs to establish an in vivo model for targeted therapy studies. While recent work has focused on understanding the role of engineered ECM properties in tumor PDO (not EAC) resistance to therapy (19, 23, 32), we showed that our in vivo xenograft model allows for identification of matrix stiffness–dependent expression of transcription factors that can be exploited for targeted therapy in EAC. Therefore, this engineered 3D organoid culture platform lays the foundation for application of therapeutics to disrupt the contribution of ECM stiffness in EAC and potentially other cancers. Taking these data together, we provide mechanistic and translational insights with broad applicability.

## Methods

### Patient-derived 3D organoid generation and culture.

We used 3 EAC PDO lines, namely EAC000, EAC006, and HNEC001, as described previously (9, 10), and a BE PDO line (BE109), as described previously (36). Briefly, the human EAC or BE tissue biopsy was digested with dispase (Corning) and trypsin-EDTA (Invitrogen), followed by mechanical dissociation and passing through a 100 μm cell strainer (Falcon). The enzymes were inactivated by soybean trypsin inhibitor (Sigma-Aldrich) and cells were washed in Dulbecco’s PBS (DPBS), counted, and seeded at 25,000 cells per 50 μL Matrigel (Corning) per 1 well of a 24-well plate. For passaging of EAC PDOs in Matrigel, the organoids were dissociated as single-cell suspension with trypsin-EDTA and seeded at 25,000 cells per 50 μL Matrigel. For passaging of BE PDOs in Matrigel, the organoids were mechanically dislodged by pipetting through a P200 pipette tip attached to a P1000 pipette tip to break down into small fragments and seeded at 50–100 fragments per 50 μL Matrigel. As previously described (9), the organoid growth medium was composed of (50%) L-WRN cell-conditioned medium expressing Wnt-3A, R-Spondin1, and Noggin (WRN), and 50% Advanced DMEM-F12 (Thermo) supplemented with 1× GlutaMAX, 10 mM HEPES, 1× N-2, 1× B-27, 1 mM NAC, 0.5 μM CHIR99021, 250 ng/mL EGF, 0.5 μM A83-01, 1 μM SB202190, 0.1 μM Gastrin, 20 mM Nicotinamide, 10 μM Y-27632, 10 μM Gentamicin, and 1× antibiotic-antimycotic. The organoid medium was refreshed every 2 to 3 days, and organoids were passaged every 7 to 14 days. For all experiments, mycoplasma-free PDO lines were limited to fewer than 15 passages.

### Hydrogel synthesis, fabrication, and PDO encapsulation.

Hydrogels were synthesized as previously described (30). Briefly, prior to NorHA macromer synthesis, sodium HA (NaHA; Lifecore Biomedical) was converted to its tetrabutylammonium salt (HA-TBA) using the Dowex 50W proton exchange resin (MilliporeSigma). To synthesize the NorHA macromer, HA-TBA was dissolved in anhydrous DMSO (2 wt%) with a 3:1 M ratio of 5-norbornene-2-carboxylic acid (mixture of endo and exo isomers; MilliporeSigma) to HA-TBA repeat units, and 4-(dimethylamino) pyridine (1.5 M ratio to HA-TBA repeat units; MilliporeSigma) was added under an N2 atmosphere. The product was analyzed by 1H NMR spectroscopy, and NorHA was found to have approximately 25% of its repeat units functionalized with norbornene. For fabrication of NorHA hydrogel, NorHA macromer (MW: 30 kDa) was dissolved in DPBS at 2% w/v. Adhesive and crosslinking peptides were custom synthesized by GenScript (https://www.genscript.com/). Adhesive peptide RGD (GCGYGRGDSPG) or RDG (GCGYGRDGSPG) was dissolved in DPBS at 50 mM (25× final ligand density). Bis-cysteine crosslinking peptide VPM (GCNSVPMSMRGGSNCG) or nondegradable crosslinking agent DTT (1,4-dithiothreitol) (Sigma, 3483-12-3) was dissolved in diH_2_O at 27.3 mM. Photo-initiator Irgacure 2959 (Ciba, I2959) was dissolved in DPBS at 0.5 wt%. For PDO encapsulation, organoids that were expanded in Matrigel for up to 14 days were retrieved by enzymatic digestion of the Matrigel using Dispase (Corning) and resuspended at ×6.67 final density (final density: 25,000 EAC PDO cells or 50 BE fragments per 25 μL hydrogel) in organoid growth medium. Hydrogel precursor solutions and PDO cell solution were mixed and photopolymerized with a curing lamp (OmniCure S1500, Excelitas Technologies) with an internal visible light filter (390 nm) at an intensity of 10 mW/cm^2^ for 5 minutes. For all in vitro experiments, 25,000 (single-cell suspension) EAC PDO cells or 50 BE fragments were encapsulated in 25 μL NorHA hydrogels unless explicitly stated in the figure legend. Sample size was established as at least 4 NorHA hydrogels per condition with the premise that an outcome present in 4 different hydrogels under a specific condition will reveal the population behavior submitted to this given condition. For all in vivo experiments, 200,000 cells (single-cell suspension of EAC PDOs) were encapsulated in 50 μL NorHA hydrogels. For in vitro experiments using verteporfin (Selleck Chemicals, S1786; 5 μM in DMSO), verteporfin was added to the culture media at a final concentration of 5 nM for the duration of the experiment. For in vitro experiments using siRNA, YAP siRNA (Santa Cruz Biotechnology Inc., sc-38637) or control (scrambled) siRNA (MilliporeSigma, SIC003) was introduced into the cells using the Lipofectamine RNAiMAX Transfection Reagent (Invitrogen, 13778150) according to the manufacturer’s instructions. YAP siRNA or control siRNA was added to the culture media at a final concentration of 10 μM. To collect organoids from hydrogels for downstream assays, we transferred the NorHA hydrogels to 1 mg/ml hyaluronidase (MilliporeSigma, H3884) or Matrigel to Dispase (Corning) and incubated at 37°C for 20 minutes to digest the hydrogel and release the organoids.

### Rheological characterization.

Storage moduli (G′) were characterized using an oscillatory shear rheometer (AR2000, TA Instruments) fitted with a 20 mm diameter cone and plate geometry and 27 μm gap. Time sweeps (0.5% strain, 1 Hz) were performed at 37°C to characterize bulk gelation upon exposure to visible light filter (390 nm) at an intensity of 10 mW/cm^2^ for 10 minutes using an OmniCure S1500 lamp (Excelitas Technologies).

### Viability assay and quantification.

NorHA hydrogels were incubated in 2 μM Calcein-AM (Life Technologies), in growth medium for 1 hour at 37°C. Samples were imaged using an Celigo Image Cytometer (Nexcelom). Quantification of viability was performed by calculating the percentage of PDOs that (at least 75% of the organoids area) stained positive for Calcein-AM using ImageJ (NIH). The results are representative of 3 independent experiments performed with 4 NorHA hydrogel or Matrigel samples per experimental group.

### Quantitative reverse-transcription PCR.

RNA isolation was achieved using the RNAqueous Phenol-Free Total RNA Isolation Kit (Invitrogen, AM1912) according to the manufacturer’s instructions. cDNA was synthesized using the Applied Biosystems High-Capacity cDNA Reverse Transcription Kit (Thermo Fisher Scientific, 43-688-13) according to the manufacturer’s instructions. Quantitative PCR was performed using the Applied Biosystems 7500 Real Time PCR System. The primer sequences used with the SYBR Green PCR Master Mix (Applied Biosystems) were as follows: *SOX9* forward sequence, ACTTGCACAACGCCGAG and *SOX9* reverse sequence, CTGGTACTTGTAATCCGGGTG; *YAP* forward sequence, AATTGAGAACAATGACGA and YAP reverse sequence, AGTATCACCTGTATCCATCTC; *TP53* forward sequence, CTTCCATTTGCTTTGTCCCG and *TP53* reverse sequence, CATCTCCCAAACATCCCTCAC; *STAT3* forward sequence, GGTACATCATGGGCTTTATC, and *STAT3* reverse sequence, TTTGCTGCTTTCACTGAATC; housekeeping gene, *YWHAZ* forward sequence, ACTTTTGGTACATTGTGGCTTCAA; and housekeeping gene, *YWHAZ* reverse sequence, CCGCCAGGACAAACCAGTAT. Gene expression of all samples was normalized to its corresponding housekeeping gene expression before normalization to control sample.

### Immunofluorescence and immunohistochemistry analysis.

For immunofluorescence or immunohistochemistry staining of paraffin sections from PDOs, xenograft implants, or human tissue samples, these were fixed with 4% (w/v) paraformaldehyde at 4°C for 4 hours to overnight. Sections were deparaffinized and heat-induced antigen retrieval was performed using 10 mM citric acid buffer (pH 6) for 15 minutes. Samples were permeabilized using 0.5% (w/v) Triton X-100 for 10 minutes and blocked with 5% donkey serum. Primary antibody incubation was performed overnight at 4°C at the dilution stated below. Secondary antibody incubation was performed for 30 minutes at 37°C at a 1:200 dilution. The following primary antibodies were used: CD44 (1:200 dilution; Cell Signaling Technology, 3570S), fibronectin/FN1 (1:100 dilution; Cell Signaling Technology, 26836S), HA-binding protein (1:100 dilution; HABP; MilliporeSigma, 385911), Ki67 (1:50 dilution; BD Biosciences, 550609), YAP (1:100 dilution; Cell Signaling Technology, 14074), Sox9 (1:20 dilution; R&D Systems, AF3075), MUC5ac (1:200 dilution; Cell Signaling Technology, 61193), CK8 (1:100, Abcam, ab53280), and E-cad (1:100, BD Biosciences, 610182). DAPI (Vector Laboratories, H-1500) was used as a counterstain for immunofluorescence, and hematoxylin stain (Leica, 3801560) was used as counterstain for immunohistochemistry. The following secondary antibodies were used: Alexa Fluor 488 donkey anti-goat (A11055), Alexa Fluor 488 donkey anti-mouse IgG (Invitrogen, A32766), and Alexa Fluor 555 donkey anti-rabbit (A32794).

### Image acquisition and quantification.

Brightfield images of PDOs were acquired using the Celigo Image Cytometer. Quantification of organoid size and density was performed using the Celigo Image Cytometer and its analytical algorithms. Acquisition of fluorescence, immunohistochemistry, and H&E images was performed using a Keyence BZ-X800 Cell Imaging Microscope. Quantification of percentages of fluorescently labeled cells (e.g., nuclear YAP^+^ cells, Figure 4E) was done using the Keyence BZ-X800 Cell Imaging Microscope and its analytical algorithms.

### Xenograft transplantation.

Single-cell suspensions of EAC PDOs were embedded in NorHA hydrogels 2 hours prior to subcutaneous transplantation in the back (flanks) of male NOD-scid IL2Rg-null (NSG) mice (Jackson Laboratory). Mice were anesthetized and sedated by intraperitoneal injection of ketamine (100 mg/kg)/xylazine (10 mg/kg) solution, and hair was removed from the back to expose skin. A small incision was made through the skin on the mouse’s right and left flanks, and the connective tissue cleared to make a small subcutaneous space (pocket) on each side. One NorHA hydrogel containing EAC cells was delivered to each subcutaneous pocket using a surgical spatula (1 implantation per pocket, 2 pockets per animal). The skin was closed using absorbable sutures. The mice were euthanized and the transplant retrieved after 4 weeks. For drug treatment experiments, 1 week after transplantation, verteporfin (Selleck Chemicals, 100 mg/kg in DMSO; maximum of 150 μL per injection) or DMSO treatment (150 μL per injection) was given every other day for 3 weeks. At the end of 3 weeks of treatment, mice were euthanized and transplant retrieved. The results are representative of 2 experiments performed with 5 mice per condition (1 organoid implanted per subcutaneous pocket). Sample size was established as 10 transplants (2 per mouse) with the premise that an outcome present in 10 different samples under a specific condition will reveal the population behavior submitted to this given condition. No statistical method was used to predetermine sample size.

### RNA-Seq and analysis.

RNA isolation was achieved using the RNAqueous Phenol-Free Total RNA Isolation Kit (Invitrogen, AM1912) according to the manufacturer’s instructions, and the isolated RNA was sent for bulk RNA-Seq at Azenta Life Sciences. For analysis, fastp (https://github.com/OpenGene/fastp) was used to trim adapter sequences. STAR 2.7.10 (https://github.com/alexdobin/STAR) was used to align trimmed fastq files to the reference genome (mm10), and Picard CollectRnaSeqMetrics module (https://gatk.broadinstitute.org/hc/en-us/articles/360037057492-CollectRnaSeqMetrics-Picard-) was used to evaluate alignments and perform initial quality control. Gene expression was quantified with HTSEQ (https://htseq.readthedocs.io/en/master/), and these data were imported into Rstudio (R 3.5) and used as input files for DESEQ2 analysis (https://bioconductor.org/packages/release/bioc/html/DESeq2.html) to determine differentially expressed genes across different conditions. Differentially expressed genes were used as input for volcano plot and Gene Ontology (GO) Enrichment Analysis (http://geneontology.org/). The plot was generated using GraphPad Prism 6.0.

### Tumor versus normal RNA-Seq analysis.

Analysis of RNA-Seq data presented in Figure 1B was performed using a previously published online tool, Gene Expression Profiling Interactive Analysis (GEPIA) (29), collecting data from TCGA and GTEx. The ESCA data set was used. The data were plotted on a log scale (log_2_(TPM + 1)) with a jitter size of 0.4. “Match TCGA normal and GTEx data” was selected.

### WES and analysis.

Genomic DNA isolation from PDOs was achieved using the QIAamp DNA Micro Kit (QIAGEN, 56304) according to the manufacturer’s instructions, and the isolated genomic DNA was sent for WES at Azenta Life Sciences. For analysis, we used fastp (https://doi.org/10.1093/bioinformatics/bty560) to trim adapter sequences from original fastq files. We then used the BWA-MEM algorithm (https://bio-bwa.sourceforge.net) to align each sample to human reference build hg38. Picard tools (https://broadinstitute.github.io/picard/) were used for sorting and to mark duplicate reads. Following all preprocessing, we used gatk4 (https://gatk.broadinstitute.org, best practices) to recalibrate base qualities using common variation (dbsnp151) and identify short variants for all samples. Common polymorphisms were flagged for downstream processing, and snpeff (https://www.ncbi.nlm.nih.gov/pmc/articles/PMC3679285/) was used to annotate.vcf files from each sample.

### Statistics.

All experiments were performed 3 or more times independently under similar conditions, except experiments shown in Figure 4 and Figure 5, which were performed twice. Plots shown are of 1 experiment representative of all independent experiments performed under similar conditions. All immunofluorescence or immunohistochemistry images shown are representative of at least 20 images that were stained and imaged for each specific marker per experimental group for each independent experiment. All statistical analyses were performed using GraphPad Prism, version 6.0. For statistical comparisons between 2 groups, Welch’s *t* test (or Mann-Whitney *U* test for nonparametric data) was used, and among more than 2 groups, 1-way ANOVA (or Kruskal-Wallis test for nonparametric data) was used. For all data, *P* < 0.05 was considered statistically significant. To ensure rigor and reproducibility, other colleagues not involved in this study masked the labels of prepared tissue slides (histology/immunofluorescence) prior to image acquisition. For all experiments, mycoplasma-free PDO lines were limited to fewer than 15 passages.

### Study approval.

Normal and EAC tissue sections from patient biopsies were obtained as paraffin-embedded tissue samples from the Molecular Pathology Shared Resource of the Herbert Irving Comprehensive Cancer Center, as approved by the Columbia University Institutional Review Board (IRB AAAS4603; PI: Julian Abrams). All methods were performed in accordance with the Columbia University IRB Committee’s regulations on human subject research. All procedures were performed at the New York Presbyterian Hospital/Columbia University Irving Medical Center. EAC and BE biopsies for PDO generation were obtained from patients undergoing diagnostic endoscopy for suspected esophageal cancer or BE at the New York Presbyterian Hospital/Columbia University Irving Medical Center, as approved by the Columbia University Institutional Review Board (IRB AAAS4603; PI: Julian Abrams), or at the Perelman Center for Advanced Medicine, as approved by the University of Pennsylvania (IRB 813841). All patients provide informed, written consent. All animal studies were conducted following protocols approved by Columbia University’s IACUC in accordance with the US Department of Agriculture (USDA) Animal and Plant Health Inspection Service (APHIS) regulations and the NIH Office of Laboratory Animal Welfare regulations governing the use of vertebrate animals.

### Data availability.

Values for all data points in graphs are reported in the Supporting Data Values file, with the exception of data in Figure 1B, which is plotted using third-party data from Gene Expression Profiling Interactive Analysis (GEPIA). For RNA-Seq analysis data, the scripts and parameters of each step will be provided upon request to the corresponding author. The RNA-Seq data set is available through the NCBI’s Gene Expression Omnibus (GEO GSE240918). For WES data analysis, the scripts and parameters of each step will be provided upon request to the corresponding author. The WES data set is available through Gene Expression Omnibus (GEO GSE240918).

## Author contributions

RCA conducted all experiments, collected data, and performed data analyses. SWK assisted with data collection and experimental analyses. KS assisted with in vivo experiments. SK performed all sequencing analyses. CL and EMP assisted with in vitro experiments and rheological measurements. GE assisted with in vitro experiments. JTG assisted with data collection. JH assisted with statistical analyses. RCA, TK, JAB, and AKR conceptualized and designed the project and experiments. RCA, JAB, and AKR wrote the manuscript.

## Supplementary Material

Supplemental data

Supporting data values

## Version 1 10/03/2023

In-Press Preview

## Version 2 12/01/2023

Electronic publication
