# Supplementary material for: Engineered hydrogel reveals contribution of matrix mechanics to esophageal adenocarcinoma and identifies matrix-activated therapeutic targets
Source: J Clin Invest. 2023 Dec 1;133(23):e168146. doi: 10.1172/JCI168146 (PMC10688988; doi:10.1172/JCI168146)
Supplement: Supplemental data [file jci-133-168146-s093.pdf]

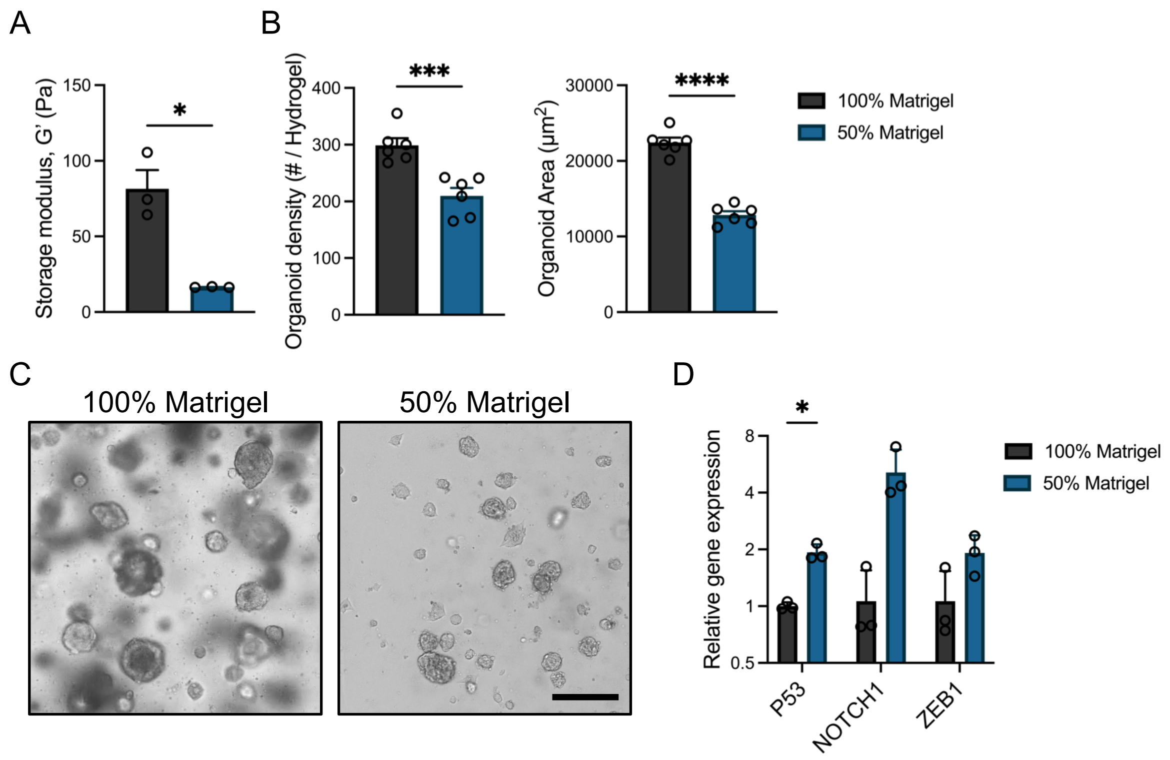

Supplementary Figure 1: EAC PDOs growth and gene expression in different Matrigel™ concentrations. (A) Relationship between Matrigel™ concentration (%) and storage modulus, G' (mean ± SEM; n = 3 independently prepared hydrogels per condition). Welch's t-test with two-tailed comparison showed significant differences between 100% and 50% Matrigel™ (*P <0.05). (B) Quantification of PDO density and size (area) as a function of Matrigel™ concentration at 10 days post-encapsulation (mean ± SEM; n = 6 hydrogels per group). Welch's t-test with two-tailed comparison showed significant differences between 100% and 50% Matrigel™ (***P <0.001, ****P <0.0001). (C) Representative transmitted light images of EAC PDOs in 100% or 50% Matrigel™. Scale bar: 250 µm. (D) Relative gene expression levels of EAC PDOs in 100% or 50% Matrigel™. RNA levels of EAC-associated genes (*TP53*, *NOTCH1*, *ZEB1*), as quantified by RT-qPCR (mean ± SEM; n = 3 samples per group), and normalized to 100% Matrigel™. Multiple Welch's t-test were used to identify statistical differences (*P <0.05). (A-D) Three independent experiments were performed and data are presented for one of the experiments. Every independent experiment was performed with six gel samples per experimental group.

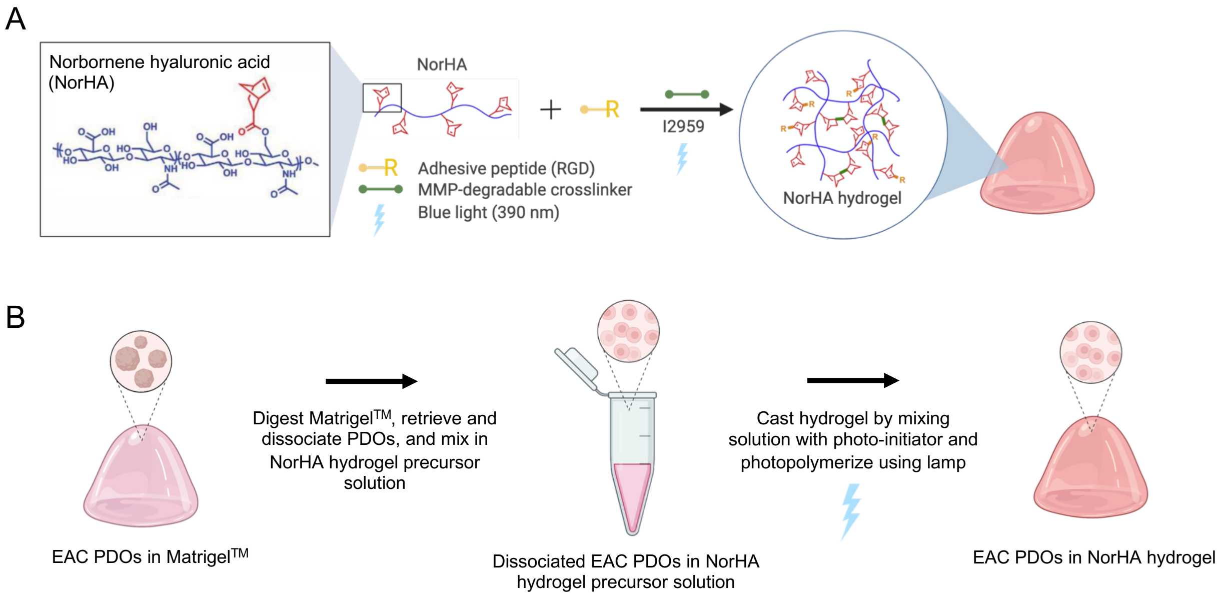

Supplementary Figure 2: NorHA hydrogel fabrication. (A) Schematic of NorHA hydrogel fabrication process to cast gels through the light-initiated thiol-ene reaction among a mono-thiol RGD adhesive ligand, a di-thiol MMP-degradable crosslinker and NorHA. Adapted from Gramlich WM, Kim IL, Burdick JA. Synthesis and orthogonal photopatterning of hyaluronic acid hydrogels with thiol-norbornene chemistry, Biomaterials (2013). (B) Schematic of EAC PDO encapsulation within NorHA hydrogel. Created with BioRender.com.

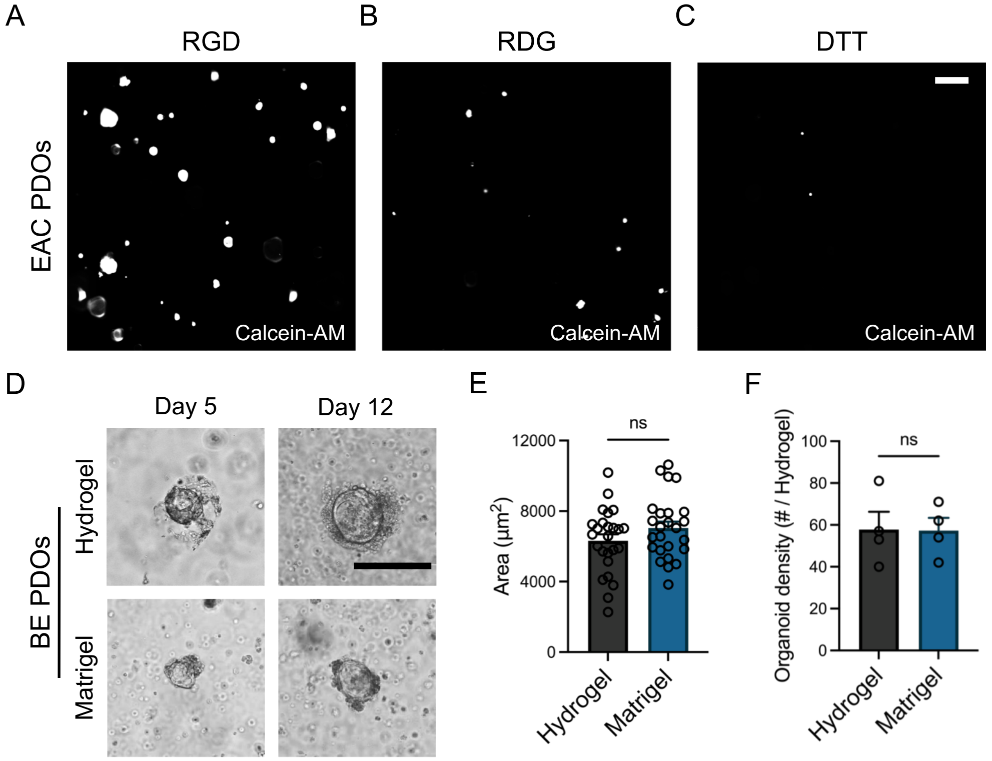

Supplementary Figure 3: Engineered NorHA hydrogel presenting RGD adhesive peptide and MMP-degradable crosslinker promote EAC PDO viability and BE PDO growth. (A-C) Representative fluorescence images of EAC PDO viability (Calcein-AM) at 7 days after encapsulation within NorHA (100 Pa) hydrogels functionalized with (A) RGD, or (B) RDG, or (C) functionalized with RGD and crosslinked with non-degradable agent DTT. Scale bar, 500 µm. (D) Representative transmitted light images and quantification of (E) size (area) and (F) formation (density) of BE PDOs cultured in engineered NorHA hydrogel or Matrigel™ (mean ± SEM; (E) n = at least 25 organoids analyzed across 4 hydrogels per group; (F) n = 4 hydrogels per group). Organoid size (area) and formation (density) were quantified after 7 days post-encapsulation. Welch's t-test with two-tailed comparison showed no significant differences between groups (ns = P > 0.05). Scale bar: 200 µm. (A-D) Three independent experiments were performed and data are presented for one of the experiments. Every independent experiment was performed with four gel samples per experimental group.

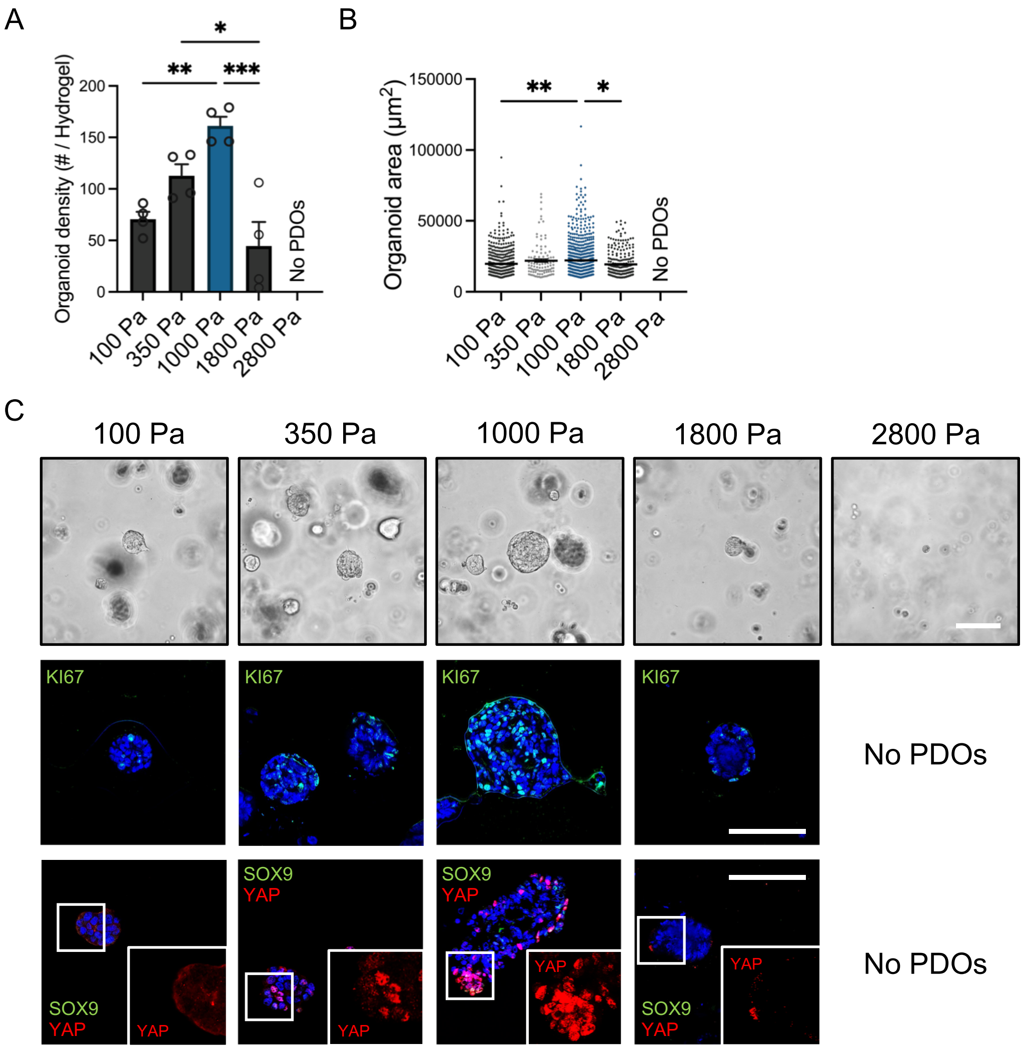

Supplementary Figure 4: Matrix stiffness-mediated growth, proliferation, and activation of the Yap-Sox9 axis in EAC PDOs. (A, B) Quantification of (A) PDO formation (density) and (B) size (area) as a function of matrix stiffness at 14 days post-encapsulation (mean ± SEM; (A) n = 4 hydrogels per group; (B) n = at least 107 organoids analyzed across 4 hydrogels per group). (A) One-way ANOVA with Tukey's multiple comparisons test showed significant differences between 100 Pa and 1000 Pa, 350 Pa and 1800 Pa, 1000 Pa and 1800 Pa (*P <0.05, **P <0.01, ***P <0.001). (B) Kruskal-Wallis test with Dunn's multiple comparisons showed significant differences between 100 Pa and 1000 Pa, 1000 Pa and 1800 Pa (*P <0.05, **P <0.01) (C)

Representative transmitted light images, and immunofluorescence staining images of EAC PDOs stained for cell proliferation (Ki67), Yap and Sox9, cultured within NorHA hydrogels of different stiffnesses at 14 days post-encapsulation. Of note, 2,800 Pa NorHA hydrogel condition did not support organoid formation. Scale bars: 100 μm. (A-C) Three independent experiments were performed and data are presented for one of the experiments. Every independent experiment was performed with four gel samples per experimental group.

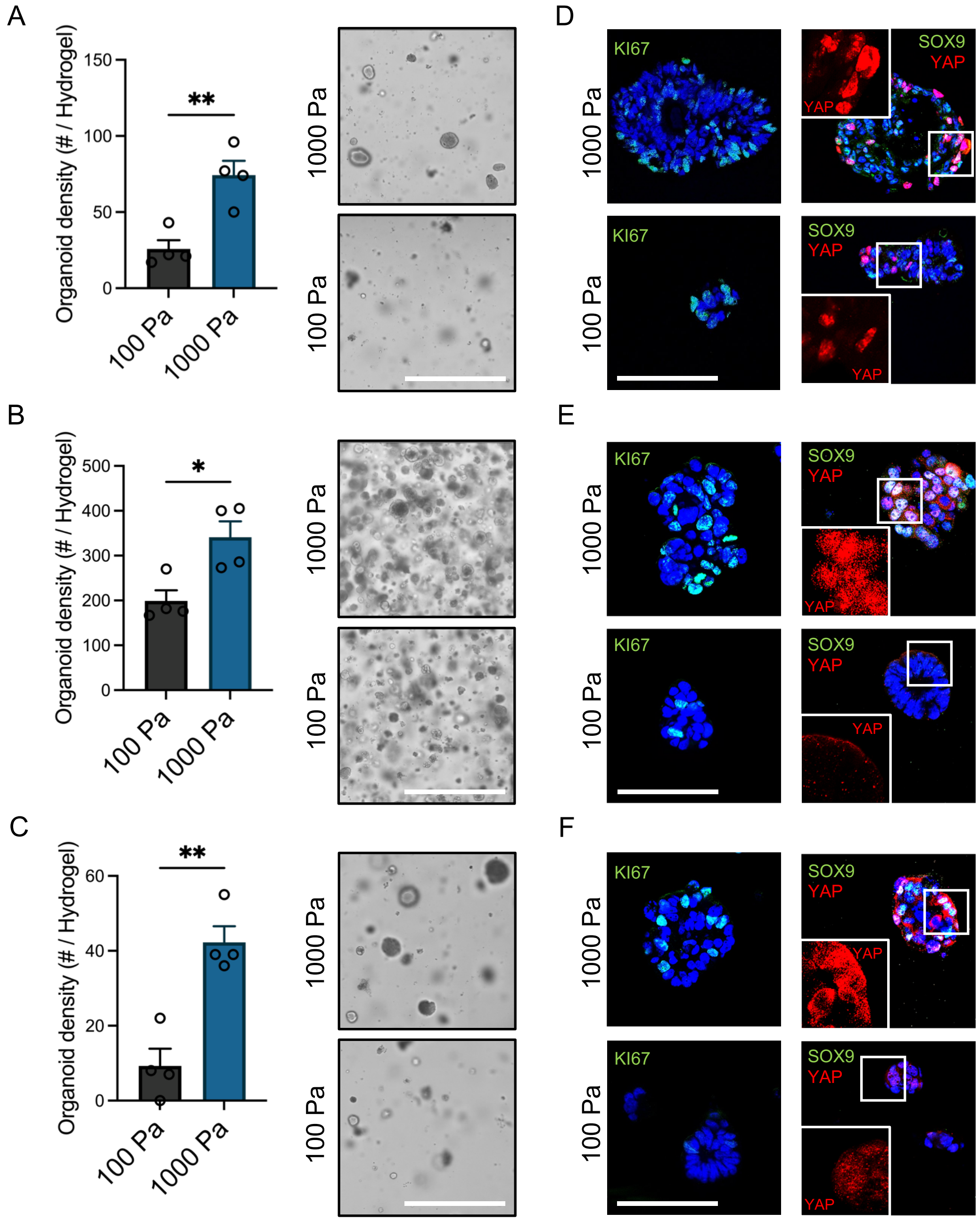

Supplementary Figure 5: Matrix stiffness-mediated growth, proliferation, and activation of the Yap-Sox9 axis in long-term culture of 3 different EAC PDOs lines. (A, B, C) Representative transmitted light images, and quantification of (A) EAC000, (B) HNEC001, and (C) EAC006 PDO formation (density) as a function of matrix stiffness at ~1.5 months post-encapsulation (after 3 passages, 14 - 15 days/passage) (mean ± SEM; n = 4 hydrogels per group).

Welch's t-test with two-tailed comparison showed significant differences between 100 Pa and 1000 Pa (*P <0.05, **P <0.01). Scale bars: 1 mm. (D, E, F) Representative immunofluorescence staining images of (D) EAC000, (E) HNEC001, and (F) EAC006 PDOs stained for cell proliferation (Ki67), Yap and Sox9, cultured within NorHA hydrogels of different stiffnesses at ~1.5 months post-encapsulation (after 3 passages, 14 - 15 days/passage). Scale bars: 100 μm. (A-F) Three independent experiments were performed and data are presented for one of the experiments. Every independent experiment was performed with four gel samples per experimental group.

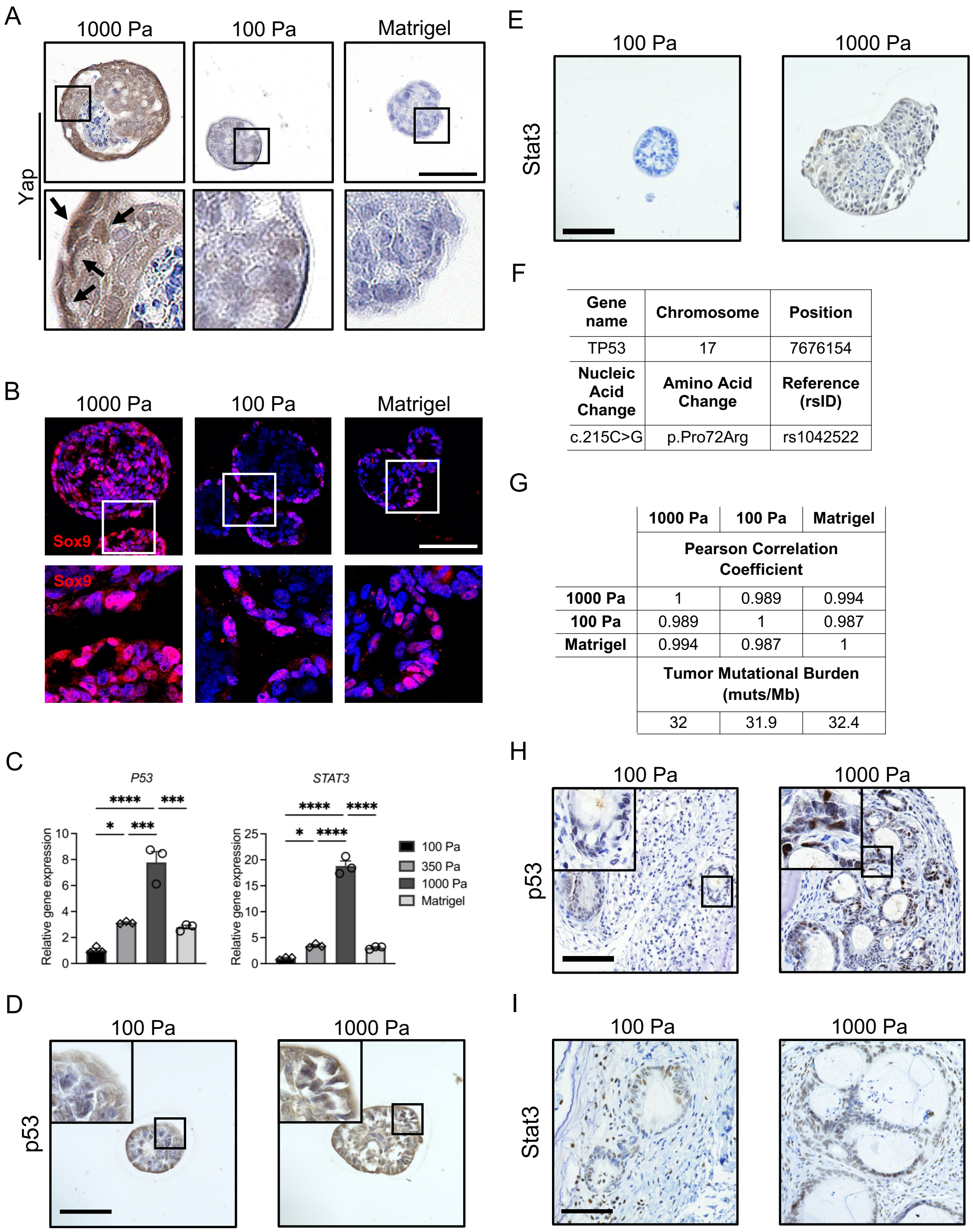

Gene name	Chromosome	Position
TP53	17	7676154
**Nucleic Acid Change**	**Amino Acid Change**	**Reference (rsID)**
c.215C>G	p.Pro72Arg	rs1042522

	1000 Pa	100 Pa	Matrigel
	Pearson Correlation Coefficient		
**1000 Pa**	1	0.989	0.994
**100 Pa**	0.989	1	0.987
**Matrigel**	0.994	0.987	1
	Tumor Mutational Burden (muts/Mb)		
	32	31.9	32.4

Supplementary Figure 6: Matrix stiffness-mediated expression of EAC-associated genes. Representative immunohistochemistry images of (A) YAP and (B) SOX9 in organoids within NorHA hydrogels of different stiffness at 14 days post-encapsulation. Arrows indicate Yap nuclear translocation. Scale bars: 100 μm. (C) Transcriptional expression of *TP53* and *STAT3* in EAC

PDOs within NorHA hydrogels of different stiffness or Matrigel™ at 14 days post-encapsulation (mean ± SEM; n = 3 technical replicates; RNA levels normalized to 100 Pa). One-way ANOVA with Tukey's multiple comparisons test showed significant differences between 100 Pa and 350 Pa, 100 Pa and 1000 Pa, 350 Pa and 1000 Pa, 1000 Pa and Matrigel™ (*P <0.05, ***P <0.001, ****P <0.0001). (D, E) Representative images of immunohistochemical (D) p53 and (E) Stat3 staining of EAC PDOs cultured within NorHA hydrogels of different stiffness at 14 days post-encapsulation. Scale bars: 100 µm. (F) Whole exome sequencing of EAC PDOs within NorHA hydrogels of different stiffness (100 or 1000 Pa) or Matrigel™ reveals presence of a *TP53* gene Pro72Arg (rs1042522) single nucleotide polymorphism. (G) Overall mutational profile of the EAC PDOs within NorHA hydrogels of different stiffness (100 or 1000 Pa) or Matrigel™ represented by Pearson Correlation Coefficient and Tumor Mutational Burden (muts/Mb), per whole exome sequencing. (H, I) Representative images of immunohistochemical (H) p53 and (I) Stat3 staining of EAC PDOs cultured within NorHA hydrogels of different stiffnesses at 28 days post-encapsulation and in vivo transplantation. Scale bars: 100 µm. (A-E) Three independent experiments were performed and data are presented for one of the experiments. Every independent experiment was performed with four gel samples per experimental group. (F, G) One independent experiment was performed with four gel samples per experimental group. (H, I) Two independent experiments were performed and data are presented for one of the experiments. Every independent experiment was performed with two gels per mouse and five mice per experimental group.

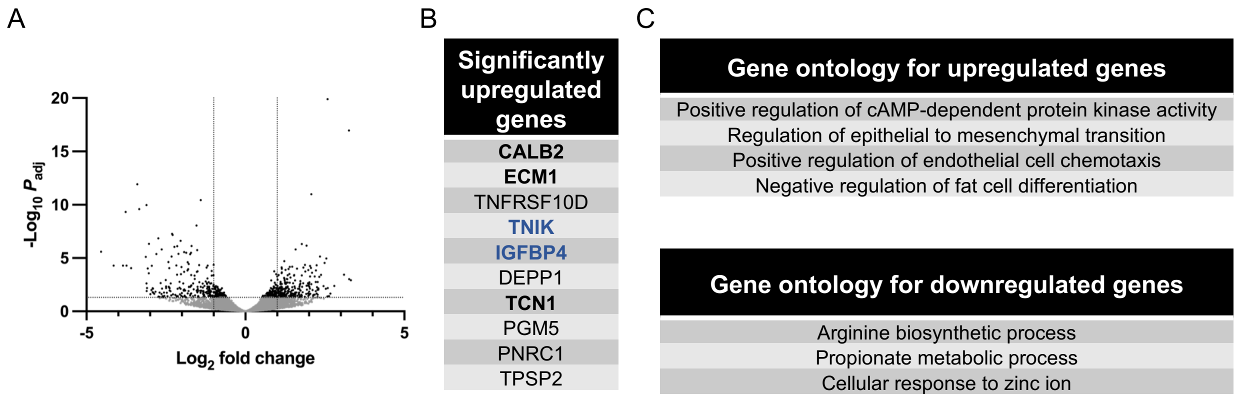

Supplementary Figure 7: Bulk RNA sequencing reveal matrix-mediated transcriptional changes in EAC PDOs. (A) Volcano plot of differentially expressed genes ($P_{adj} < 0.05$ and |log2 fold change| > 1), as profiled by RNA-sequencing, in EAC PDOs within stiff NorHA hydrogel versus Matrigel™ resulted in 424 differentially expressed genes (224 upregulated and 200 downregulated genes; Yap was upregulated but did not show significance). (B) List of top 10 significantly upregulated genes in EAC PDOs within stiff NorHA hydrogel versus Matrigel™ (P_{adj} < 0.05 and |log2 fold change| > 1). Gene names in bold have been correlated with tumor progression, metastasis, or recurrence. Gene names in bold and blue color are downstream targets of Yap and have been correlated with tumor progression. (C) Gene ontology of up- and down-regulated genes showing enrichment of biological processes in EAC PDOs within stiff NorHA hydrogel versus Matrigel™.

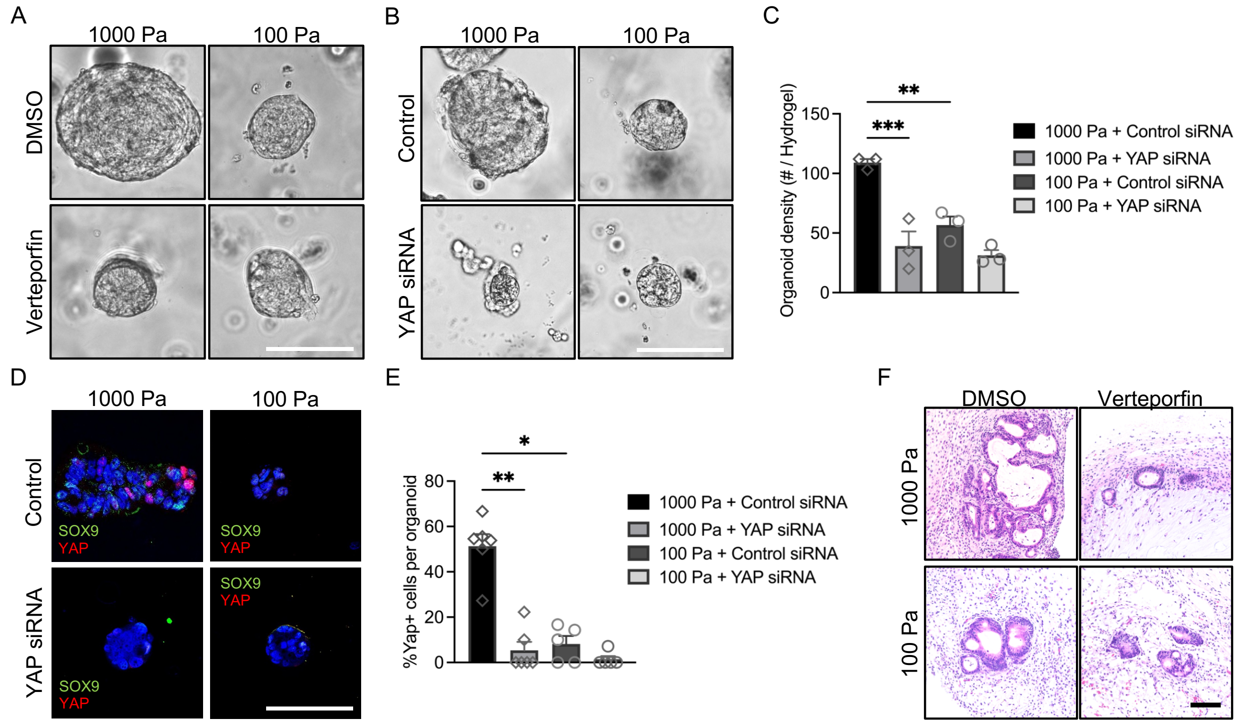

Supplementary Figure 8: NorHA hydrogel as a platform for targeted therapy studies. (A, B) Representative phase contrast images of EAC PDOs cultured in NorHA hydrogels of different stiffnesses at 7 days post-encapsulation and treated with (A) 5 nM Verteporfin or DMSO, or (B) 10 μM YAP siRNA or control (scrambled) siRNA. Scale bars: 100 μm. (C) Quantification of PDO formation (density) within NorHA hydrogels of different stiffnesses at 7 days post-encapsulation and treated with 10 μM YAP siRNA or control (scrambled) siRNA (mean ± SEM; n = 3 hydrogels per group). One-way ANOVA with Sidak's multiple comparisons test showed significant differences between 1000 Pa + Control siRNA and 100 Pa + Control siRNA (**P <0.01), 1000 Pa + Control siRNA and 1000 Pa + Yap siRNA (***P <0.001), and no significant differences between 100 Pa + Control siRNA and 100 Pa + Yap siRNA (P > 0.05). (D) Representative fluorescence microscopy images of EAC PDOs cultured in NorHA hydrogels of different stiffnesses at 7 days post-encapsulation and treated with 10 μM YAP siRNA or control siRNA and stained for Yap and Sox9. Scale bar: 100 μm. (E) Quantification of percentage of nuclear Yap+ cells per EAC PDO cultured in NorHA hydrogels of different stiffnesses at 7 days post-encapsulation and treated with

10 μM YAP siRNA or control siRNA (mean ± SEM; n = at least 5 organoids analyzed across 4 hydrogels per group). Kruskal-Wallis test with Dunn's multiple comparisons showed significant differences between 1000 Pa + Control siRNA and 100 Pa + Control siRNA (*P <0.05), 1000 Pa + Control siRNA and 1000 Pa + Yap siRNA (**P <0.01), and no significant differences between 100 Pa + Control siRNA and 100 Pa + Yap siRNA (P > 0.05). (F) Histological (H&E) microcopy images of PDOs within NorHA hydrogels at 28 days post-encapsulation, in vivo transplantation, and treatment with Verteporfin or DMSO. Scale bar: 100 μm. (A-D) Three independent experiments were performed, and data are presented for one of the experiments. Every independent experiment was performed with four gel samples per experimental group. (E) Two independent experiments were performed, and data are presented for one of the experiments. Every independent experiment was performed with two gels per mouse and five mice per experimental group.
